# Factorial design study of self-management using Dnurse App in T2DM patients

**DOI:** 10.3389/fendo.2025.1420578

**Published:** 2025-03-28

**Authors:** Hongxia Tang, Huiwen Tan, Jihong Zhang, Mingjiao Zhang, Mengjie Chen, Laixi Kong, Xiaoxia Wang, Maoting Guo, Jinxiu Zhao, Lili Song, Zijun Zheng, Huiqi Yang, Zhe Li, Zhenzhen Xiong

**Affiliations:** ^1^ School of Nursing, Chengdu Medical College, Chengdu, Sichuan, China; ^2^ Department of Neurology, The First Affiliated Hospital of Chengdu Medical College, Chengdu, Sichuan, China; ^3^ Department of Endocrinology and Metabolism, West China Hospital, Sichuan University, Chengdu, Sichuan, China; ^4^ The School Hospital, Southwest Petroleum University, Chengdu, Sichuan, China; ^5^ Department of Nursing, Nanbu People’s Hospital, Nanchong, Sichuan, China; ^6^ Mental Health Center, West China Hospital, Sichuan University, Chengdu, Sichuan, China

**Keywords:** type 2 diabetes mellitus, diabetes app, self-management, factorial design trial, Chinese, blood glucose monitoring

## Abstract

**Background:**

With the popularity of smart phones and the development of information technology, more and more patients are adopting diabetes APPs for self-management. However, at present, there are few research reports on the effect of those APPs coming from China.

**Objective:**

The purpose of this study was to evaluate the effectiveness and applicability of an APP for blood glucose control that is widely popular among Chinese patients with type 2 diabetes mellitus (T2DM).

**Methods:**

This is a 2-center, factorial design, with equal proportional distribution, and superiority trial conducted in outpatient endocrinology clinics at two tertiary hospitals in Chengdu, China. The trial enrolled smartphone-literature individuals aged at least 18 years old who have been diagnosed with T2DM based on glycosylated hemoglobin A_1c_ (HbA_1c_) of at least 7.0%. The subjects were randomly divided into 4 groups, which were the usual care group (G1); the telephone follow-up group (G2); the APP group (G3); the APP & telephone follow-up group (G4). After 6 months of these interventions, the primary outcome was HbA_1c_, and the secondary outcomes were blood pressure (BP), body mass index (BMI), frequency of self-monitoring of blood glucose (SMBG), and satisfaction with the APP.

**Results:**

273 patients with type 2 diabetes were included in the study, among which 226 (82.8%) were followed up at the 3rd month and 220 (80.6%) at the 6th month. There was no significant difference in HbA_1c_ attainment rate among the four groups after intervention (*P* >.05), but the HbA_1c_ attainment rate in the two APP groups was higher than that in the other groups. The systolic blood pressure (SBP) of the two APP groups was significantly lower than that of the other groups (*P* <.05). There was no significant difference in the compliance rate of SMBG among the four groups (*P* >.05). Each item of the participants’ satisfaction evaluation of the APP scored more than 4.5 points.

**Conclusions:**

The diabetes APP has a tendency to improve the HbA_1c_ compliance rate of T2DM patients. The APP can help reduce patients’ BP, and patients have a high satisfaction evaluation of the APP. Therefore, the study supports the use of the APP for self-management in people with type 2 diabetes.

**Clinical Trial Registration:**

https://www.chictr.org.cn, identifier ChiCTR2100042297.

## Introduction

Over the past 30 years, China has witnessed a significant increase in the prevalence of diabetes, driven by rapid urbanization, an aging population, a rise in overweight and obesity rates, among other factors (1). Consequently, China now has the largest number of diabetes patients in the world (2). The widespread prevalence of diabetes imposes a heavy economic burden and substantially impacts patients’ quality of life (2). Therefore, it is crucial to urgently enhance patients’ self-management ability to improve the prognosis and outcome of the disease. Unfortunately, the level of self-management among diabetics in China remains less than ideal (3, 4).

In recent years, as communication technology has advanced and smartphones have become increasingly popular, a growing number of individuals with diabetes are turning to mobile apps for assistance in managing their condition. Relevant studies have reported the effectiveness of diabetes apps in improving patients’ self-management (5) and metabolic control (6, 7). However, there is a lack of research focusing on this topic in China, particularly in Chengdu, a western city with a less developed economy. The status quo of app usage and its potential impact on improving blood glucose levels among patients remain unclear.

The intervention tool selected for this study, the Dnurse App, stands out as the most popular among T2DM patients, according to a survey conducted across 30 provincial-level regions in China (8). The Dnurse smart blood glucose meter allows for continuous monitoring of blood glucose levels, automatically transmitting this data to the app where it is stored. The app also provides detailed reports, allowing users to track the total number of blood glucose tests conducted throughout the current week, month, and quarter. The app’s background can calculate the simulated HbA_1c_ based on a patient’s blood glucose values, allowing for clear visualization of blood glucose changes. Additionally, through the Intelligent Decision Support System, the app comprehensively analyzes users’ blood glucose, diet, exercise, and other data to provide personalized reminders, recommendations, encouragement, and interaction, thereby facilitating individualized diabetes management.

## Methods

### Study design

**Figure 1:**
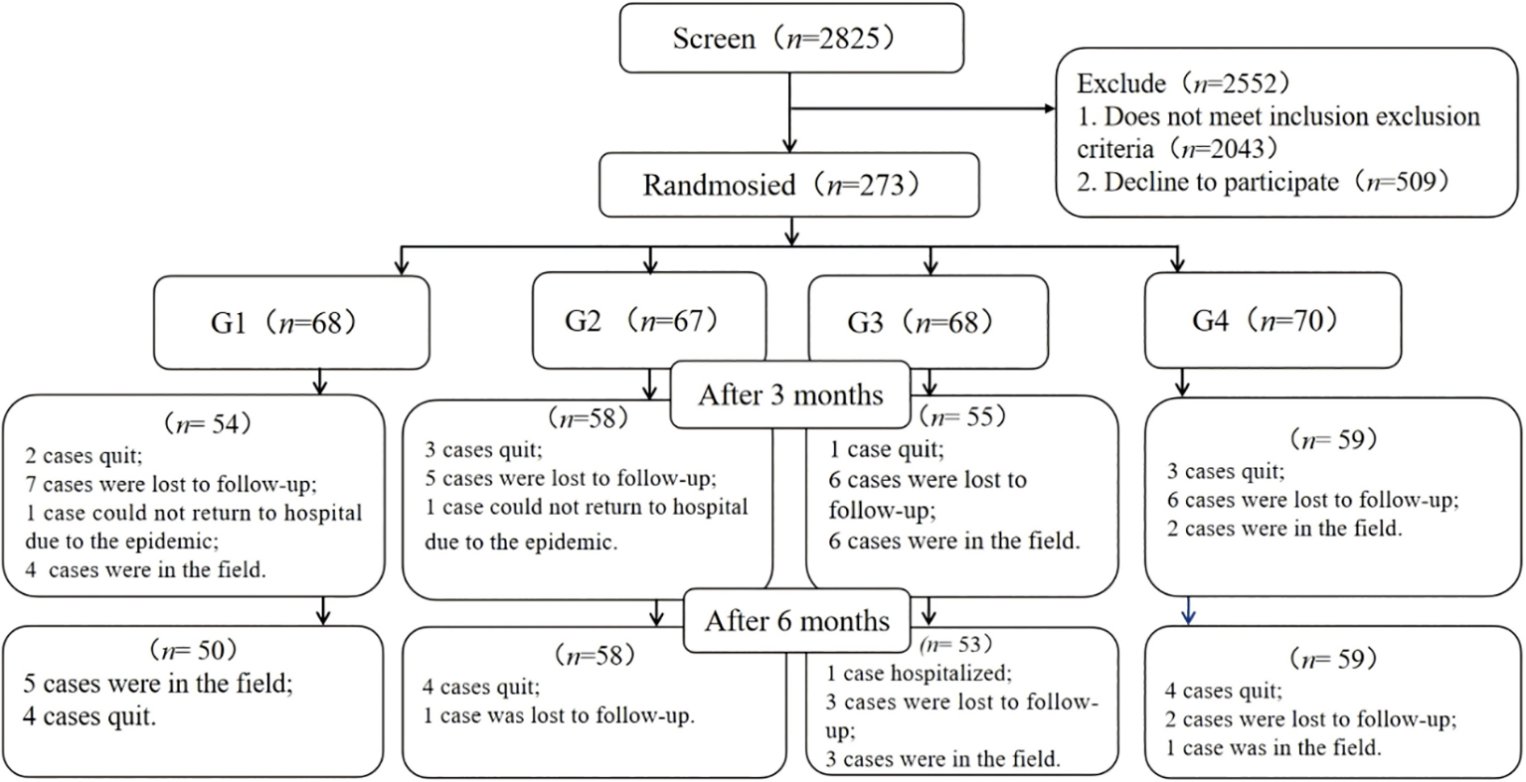
Flowchart of participant enrollment and status. G1, usual care; G2, telephone follow-up; G3, App; G4, App & telephone follow-up.

Utilizing a 6-month, open-label, parallel-group, four-factorial design, this study was conducted at two affiliated hospitals of a university located in Chengdu, Sichuan Province, China from October 2021 to August 2023. Participants were randomly assigned to one of four groups: the usual care group (G1), the phone follow-up group (G2), the app group (G3), or the app & phone follow-up group (G4). Detailed research protocols have been previously published (9). Out of 2,825 individuals screened, 273 or 9.7% were enrolled in the study. Out of those enrolled, 220 or 80.6% completed the study six months after the intervention (see **Figure 1**).

### Study patients

The inclusion criteria for this study are as follows: individuals diagnosed with T2DM by a secondary or higher-level hospital according to the World Health Organization guidelines; individuals with an HbA_1c_ level of 7.0% or higher; individuals who have completed at least primary school education; individuals who are 18 years of age or older; individuals who are proficient in using smartphones (assessed by having installed at least three commonly used apps on their device); individuals who have resided in Chengdu for at least the past 12 months; and individuals who are willing to participate in the study and provide informed consent.

Exclusion criteria are as follows: individuals who have previously used a diabetes-related app; individuals with a self-reported history of mental illness, cognitive impairment, or communication disorders; individuals who are pregnant or planning to become pregnant; individuals with any severe, life-threatening diseases; and individuals currently hospitalized for diabetes-related reasons.

### Ethical approval

The study received ethical approval from the Ethics Committee of the First Affiliated Hospital of Chengdu Medical College (2020CYFYIRB-BA-129-F01). Prior to participating in the study, all individuals provided written informed consent. The research was conducted in compliance with the principles outlined in the Declaration of Helsinki.

### Sample size

In this experiment, a factorial design is employed, and the necessary parameters are not available from previous studies. Utilizing GPower 3.1 (University of Kiel, Kiel, Germany), we calculated the required sample size based on the anticipated outcomes. Assuming a small effect size of 0.2 (10), with a two-sided α level of 0.05, and a test power (1-β) of 0.9, we used an F-test, resulting in a sample size of 220 cases. To account for an estimated 20% attrition rate, the total sample size was increased to 264 cases.

### Randomization

Prior to the commencement of enrollment, two sets of random numbers were generated using Excel by individuals not involved in the study. Each set was assigned a serial number in advance and placed in an opaque, sequentially numbered envelope. After the baseline assessment was completed, participants were randomly allocated to G1, G2, G3, or G4 in equal proportions (1:1:1:1).

### Interventions

G1 participated in conventional diabetes management. They were provided with the Dnurse He pro wisdom, a standard portable glucose meter developed by Beijing Dnurse Technology Co., Ltd. based in Beijing, China, and a blood glucose monitoring logbook. According to national guidelines, they were instructed on the appropriate timing and frequency for monitoring their blood glucose levels (11).

G2 participants received the same materials as G1, along with weekly phone reminders to consistently self-monitor their blood glucose levels. These reminders were delivered by carefully chosen nursing undergraduates who had been trained in the study’s rationale, interventions, and objectives. Only those students who excelled in post-training evaluations were selected to participate in the study. The phone follow-up was conducted in a structured manner and involved two questions. The first question asked, “Have you monitored your blood glucose regularly this week?” If the response was “no,” the second question was, “Could you please explain why you haven’t monitored your blood glucose regularly?”

The participants in G3 were provided with the Dnurse SPUG mobile blood glucose and uric acid tester, a smart blood glucose meter developed by Beijing Dnurse Technology Co., Ltd. Under the guidance of a specially trained researcher, they downloaded the Dnurse App 4.0.16 (accessible at dnurse.com/v2/app), which is specifically designed to integrate seamlessly with the smart glucose meter. The app is compatible with both Android and iOS operating systems. Based on the essential goal of boosting patients’ autonomous compliance, the Intelligent Decision Support System is used to perform intelligent matching.

**Figure 2:**
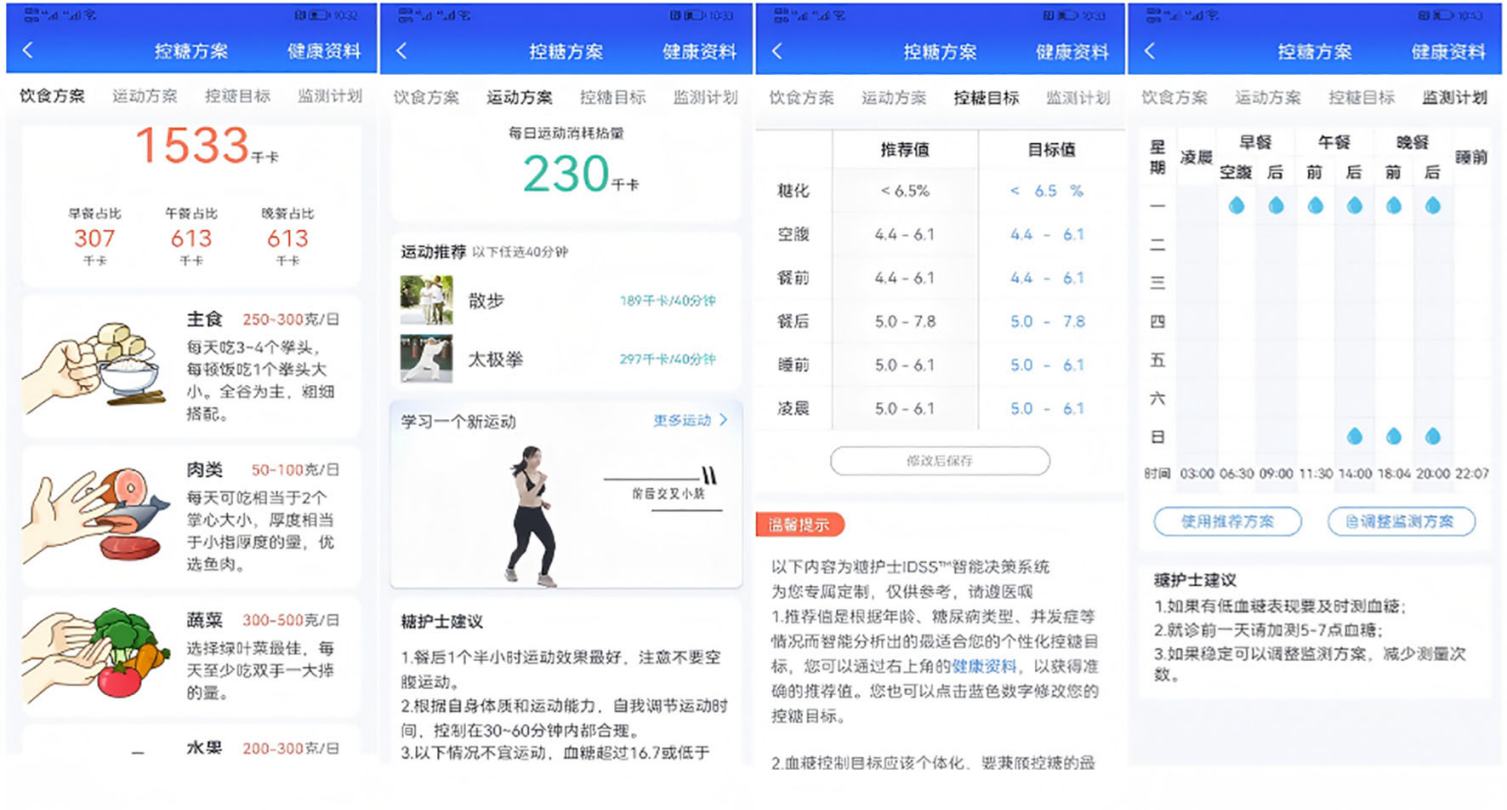
Blood glucose control plan.

**Figure 3:**
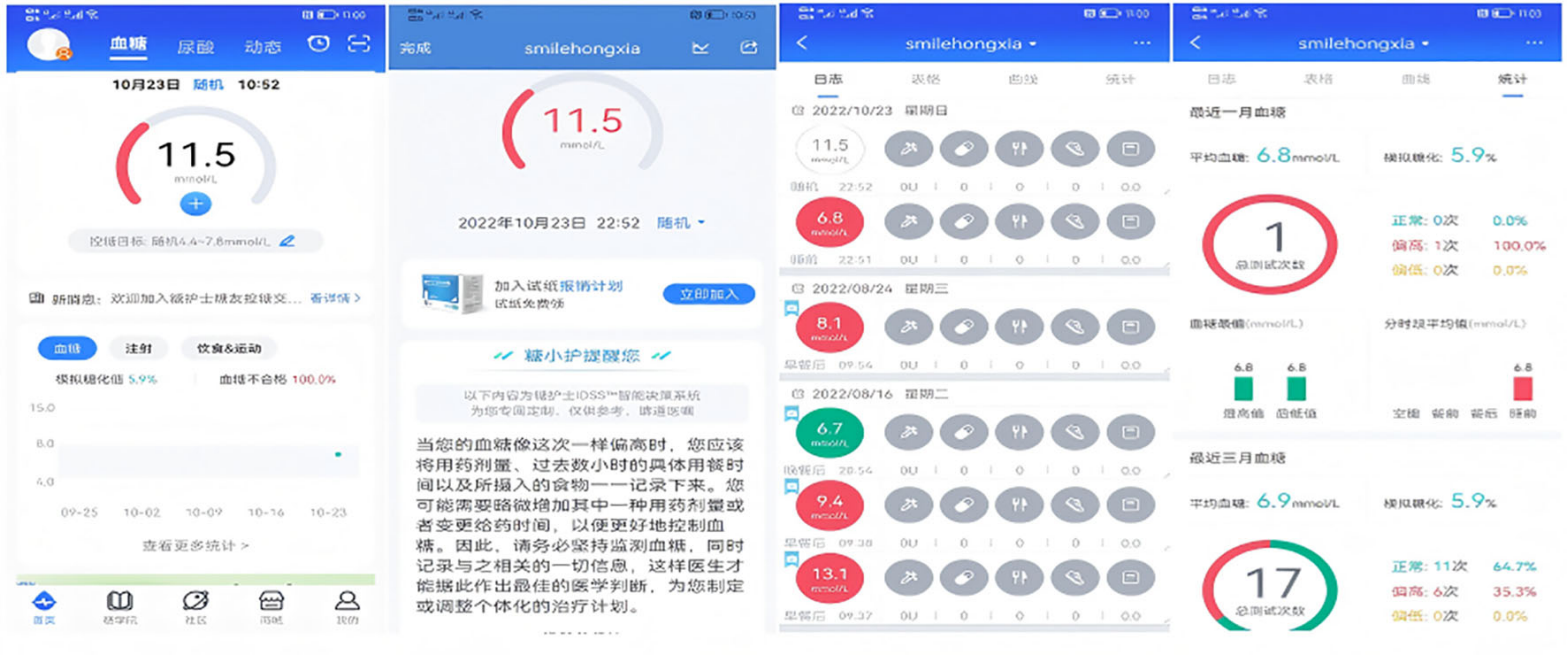
Blood glucose data management and analysis.

**Figure 4:**
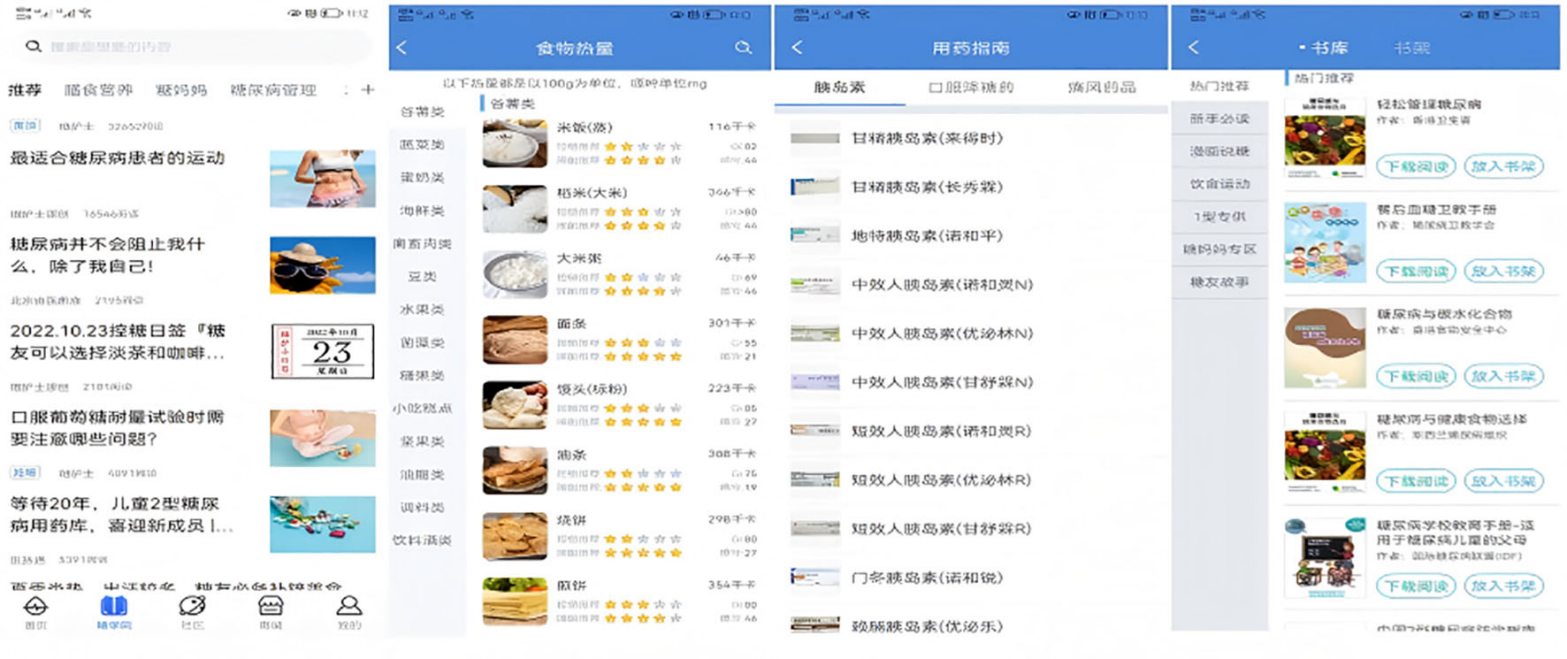
Blood glucose control knowledge.

Participants were guided through the process of setting up a personal account on the app. They were also instructed on how to create customized diet and exercise plans, establish blood glucose control goals, and develop a blood glucose monitor plan. Additionally, they learned how to access features such as food banks, disease information, real-time blood glucose analysis, and recommendations available within the app. Users were also shown how to set reminders for taking medication and managing other behaviors. To remind the same user about the same matter, the app’s Intelligent Decision Support System draws on over 6,000 scenarios to ensure that every reminder feels like a “human greeting” rather than “machine-generated text” (see **Figures 2**–**4**).

G4 received the same materials and training as G3, along with weekly phone reminders similar to those given to G2.

When participants in G1 and G2 encountered acute complications of diabetes, such as extremely high or low blood glucose levels, clinical trial investigators would recommend seeking medical treatment according to standard procedures. When participants in G3 and G4 triggered a “critical value” alarm on the app, the manufacturer’s customer service staff would contact them by phone to offer guidance or information following standard procedures. After the study, to compensate participants in G1 and G2, we introduced the Dnurse App to them and provided instructions on its use. Both devices have obtained ISO13485 and EU CE certifications.

### Primary and secondary outcomes

The primary outcome measured was the difference in the change of HbA_1c_ levels (%) from the baseline to the sixth month across the four groups. The HbA_1c_ level <7% attainment rates were evaluated at both the third and sixth months. The secondary outcomes included blood pressure (BP), body mass index (BMI), and self-monitoring of blood glucose (SMBG) compliance rate. The SMBG compliance rate was defined according to the 2009 Guidelines for Diabetes Care and Education in China as monitoring blood glucose at least once a day for patients using insulin and at least once a week for those not using insulin (12).

Participant satisfaction in G3 and G4 was evaluated at the sixth month using a tailored satisfaction survey. This survey included 5-point Likert scale questions assessing various aspects of the Dnurse App and the glucose meter. Participants rated their satisfaction with the app’s user interface, the method of recording blood glucose levels in the app, and the ease of operation of both the app and the glucose meter. The 5 points denoted a response of “very satisfied” or “strongly agree,” and higher scores indicated greater satisfaction. The content validity index of the questionnaires was 0.85, and the Cronbach’s α coefficient of the scale was 0.961. All questionnaires were filled out online via Questionnaire Star.

### Measurements

The demographic and clinical information collected at both the baseline and follow-up has been detailed in previous studies (9). To ensure consistency across results, measurements of laboratory parameters, including HbA_1c_ levels (%), were made using high-performance liquid chromatography with specialized reagents at the same certified external laboratory, and the analysis was performed using the MQ-6000 HbA_1c_ analyzer, manufactured by Shanghai Huizhong Medical Technology Co., Ltd. Additionally, BP and BMI were recorded at every visit.

Hypoglycemic events—comprising hospitalizations or emergency room visits due to hypoglycemia (blood glucose levels below 3.9 mmol/L), or related symptoms even in the absence of SMBG—were assessed at the baseline and six months after intervention. Diabetes management behaviors, such as SMBG frequency, were recorded at each visit. The SMBG frequency referred to the total number of tests conducted over a three-month period. This was determined for G1 and G2 using the entries in their blood glucose monitoring logbooks, and for G3 and G4 based on the records in the app. Additionally, user satisfaction with the mobile app was surveyed for G3 and G4.

### Statistical analysis

The analysis was performed using SPSS 23.0 (IBM, Chicago, IL, USA). Continuous variables were presented as mean (SD) and M (*Q1, Q3*), whereas categorical data were reported as frequencies with percentages. To evaluate the differences among the four groups, a variety of statistical tests were employed depending on the data type: the chi-squared test for categorical data, analysis of variance (ANOVA) for normally distributed continuous data, and the non-parametric rank-sum test for skewed continuous data. To assess the differences across various time points within each group, the generalized estimating equation (GEE) approach was utilized. These models included intervention variables, time as a categorical variable with dummy coding, interactions between intervention and time, and the baseline values. A *P* value of less than 0.05 was considered to indicate statistical significance.

## Results

### Participants flow

Between October 2021 and August 2023, a total of 2,825 individuals were evaluated for eligibility at the outpatient clinics of two university-affiliated diabetes centers. Of these, 273 participants (9.7%) were randomized into the study. After a 6-month intervention, 220 participants (80.6%) remained in the study (see **Figure 1**), with an equal distribution across the groups.

### Clinical characteristics of participants

The baseline analysis revealed no significant differences between participants who completed the study and those who were lost to follow-up (all *P*>.05; **Table 1**). The mean age of the participants was 56.3 (12.4) years, 63.0% (172/273) were male, and 61.9% (169/273) had an education level of junior high school or below. The mean baseline HbA_1c_ level and duration of diabetes were 8.2% (7.2%, 9.7%) and 7.8 (6.9) years, respectively. The mean BMI was 25.0 (4.3) kg/m^2^. Additionally, more than half of the participants (58.6%) were using only oral hypoglycemic agents, and a majority (60.8%) had chronic disease complications.

**Table 1:** Baseline demographic and clinical characteristics of participants.

	G1^a^(n=68)	G2^b^(n=67)	G3^c^(n=68)	G4^d^(n=70)	*P* value
Age (years), n (%)					.122
≤60	46 (67.7)	38 (56.7)	51 (75.0)	50 (71.4)	
>60	22 (32.3)	29 (43.3)	17 (25.0)	20 (28.6)	
Sex, n (%)					.121
Male	42 (61.8)	37 (55.2)	41 (60.3)	52 (74.3)	
Female	26 (38.2)	30 (44.8)	27 (39.7)	18 (25.7)	
Education, n (%)					.173
Primary school	21 (30.9)	18 (26.9)	10 (14.7)	15 (21.4)	
Junior high school and technical secondary school	30 (44.1)	22 (32.8)	27 (39.7)	26 (37.1)	
High schools and junior college	14 (20.6)	18 (26.9)	19 (27.9)	16 (22.9)	
University and above	3 (4.4)	9 (13.4)	12 (17.7)	13 (18.6)	
Duration of diabetes (years), n (%)					.204
<5	23 (33.8)	24 (35.8)	34 (50.0)	26 (37.1)	
≥5	45 (66.2)	43 (64.2)	34 (50.0)	44 (62.9)	
HbA_1c_ (%) , M (*Q1,Q3*)	8.4 (7.3, 10.0)	8.0 (7.3, 9.4)	8.4 (7.2, 9.9)	7.9 (7.1, 9.5)	.688
BP (mm Hg), mean (SD)					
SBP^e^	133.8 (16.3)	132.6 (17.6)	127.0 (16.2)	130.4 (19.7)	.120
DBP^f^	78.4 (12.5)	80.1 (11.6)	78.9 (10.1)	79.0 (11.7)	.858
BMI (kg/m^2^), mean (SD)	24.9 (4.4)	24.4 (3.4)	25.7 (5.4)	25.2 (3.9)	.360
Treatment plan, n (%)					.502
Non-drug therapy	3 (4.4)	4 (6.0)	5 (7.3)	1 (1.4)	
Oral agents	34 (50.0)	38 (56.7)	44 (64.7)	44 (62.9)	
Insulin only	4 (5.9)	2 (3.0)	2 (3.0)	4 (5.7)	
Insulin + oral agents	27 (39.7)	23 (34.3)	17 (25.0)	21 (30.0)	
Complication, n (%)					.081
1-3 kinds	41 (60.3)	40 (59.7)	36 (52.9)	30 (42.9)	
More than 3 kinds	10 (14.7)	1 (1.5)	4 (5.9)	4 (5.7)	
Acute complications^g^, n (%)					.126
Moderate ketoacid	3 (4.4)	1 (1.5)	2 (2.9)	2 (2.9)	
Hypoglycemia	21 (30.9)	16 (23.9)	9 (13.2)	20 (29.4)	

^a^G1, usual care; ^b^G2, telephone follow-up; ^c^G3, App; ^d^G4, App & telephone follow-up; ^e^SBP, systolic blood pressure; ^f^DBP, diastolic blood pressure; ^g^Per participant who experienced acute complications for 6 months.

### Primary study outcomes: HbA_1c_ attainment rates

The HbA_1c_ level <7% attainment rate increased in G3 and G4 at the third month, but the difference was not significant (G1 vs G2 vs G3 vs G4: 32.7% vs 35.1% vs 42.0% vs 47.3%). Compared with the other groups, G3 and G4 always had higher HbA_1c_ level <7% attainment rates at the sixth month (G1 vs G2 vs G3 vs G4: 34.7% vs 33.3% vs 44.0% vs 45.5%). This indicates that there was no significant difference in the main effect of the intervention on the HbA_1c_ compliance rate (*P*=.787, **Table 2**).

**Table 2:** Changes in HbA_1c_ level <7% attainment rate.

	Time	G1^a^(*n*=49)	G2^b^(*n*=57)	G3^c^(*n*=50)	G4^d^ (*n*=55)	Main effect of intervention	Time effect	Interaction effect
						*P* value	*P* value	*P* value
HbA_1c_ attainment rates,n (%)	0M^e^	0 (0.0)	0 (0.0)	0 (0.0)	0 (0.0)	.787	<.001	.142
	3M^f^	16 (32.7)	20 (35.1)	21 (42.0)	26 (47.3)			
	6M^g^	17 (34.7)	19 (33.3)	22 (44.0)	25 (45.5)			

^a^G1, usual care; ^b^G2, telephone follow-up; ^c^G3, App; ^d^G4, App & telephone follow-up; ^e^0M, baseline; ^f^3M, 3 months after intervention; ^g^6M, 6 months after intervention.

### Secondary study outcomes: BP, BMI, and SMBG attainment rates

The main effect of the intervention on systolic blood pressure (SBP) was statistically significant (*P*<.05). Pairwise comparisons revealed that after the 3-month intervention, the SBP in G3 and G4 was lower than that in G1, and G3 was lower than G2 (**Table 3**). The results of factorial analysis showed no interaction between phone follow-up and app (*P*>.05; **Table 4**), indicating that the app effectively reduces SBP levels. However, the change in BMI levels did not significantly differ among the four groups during the 6-month intervention period. Additionally, the changes in BMI and SMBG compliance rate showed no significant differences among the study groups throughout the intervention period.

**Table 3:** Secondary study outcomes at baseline and follow-up.

	Time	G1^a^(*n*=49)	G2^b^(*n*=57)	G3^c^(*n*=50)	G4^d^ (*n*=55)	Main effect of intervention	Time effect	Interaction effect
						*P* value	*P* value	*P* value
SBP (mmHg), mean (SD)	0M^e^	134.2 (15.0)	132.8 (18.3)	126.1 (15.5)	131.0 (17.9)	.016	<.001	.113
	3M^f^	131.9 (18.8)	130.6 (14.6)	123.6 (14.8)^h,i^	124.8 (18.7)^h^			
	6M^g^	124.6 (14.3)	121.1 (15.8)	117.1 (16.7)	123.9 (16.0)			
BMI (kg/m^2^), mean (SD)	0M	24.6 (4.6)	24.5 (3.5)	25.5 (6.0)	25.1 (3.9)	.692	<.001	.184
	3M	23.4 (3.0)	23.6 (2.1)	24.0 (3.3)	23.3 (2.6)			
	6M	25.2 (7.1)	23.8 (2.3)	24.5 (3.5)	23.9 (2.9)			
SMBG attainment rates, n (%)	3M	35 (71.4)	44 (77.2)	35 (70.0)	45 (81.8)	.221	.411	.918
	6M	34 (69.4)	44 (77.2)	29 (58.0)	40 (72.7)			

^a^G1, usual care; ^b^G2, telephone follow-up; ^c^G3, App; ^d^G4, App & telephone follow-up; ^e^0M, baseline; ^f^3M, 3 months after intervention; ^g^6M, 6 months after intervention; ^h^ means compared with G1, P < .05; ^i^means compared with group G2, *P* < .05.

**Table 4:** Factorial analysis of SBP.

Source	Type III Sum of Squares	df	Mean Square	*P* value
Telephone follow-up	170.289	1	170.289	.439
APP	1303.919	1	1303.919	.033
Interaction(telephone follow-up×APP)	511.924	1	511.924	.180

The SMBG attainment rates did not differ significantly among the four groups at the 6-month follow-up (*P*>.05). However, compared with participants in G1 and G3, those in the two phone follow-up groups (G2 and G4) tended to have higher SMBG attainment rates at both the 3-month mark (G1 vs G2 vs G3 vs G4: 71.4% vs 77.2% vs 70.0% vs 81.8%) and the 6–month mark (G1 vs G2 vs G3 vs G4: 69.4% vs 77.2% vs 58.0% vs 72.7%).

### Satisfaction with Dnurse App

Out of 112 participants, 110 (98.3%) completed the satisfaction survey at the sixth month of the intervention. Participants’ satisfaction with the Dnurse App was very high in both G3 and G4. Overall, the majority of patients were satisfied with the blood glucose recording method. The second highest-rated aspect was managing diabetes using the app and its user interface (**Table 5**).

**Table 5:** Satisfaction for Dnurse APP.

Item	Score
APP	
The blood glucose recording method, mean (SD)	4.71 (0.53)
Manage diabetes with the APP, mean (SD)	4.65 (0.6)
APP interface, mean (SD)	4.65 (0.6)
Ease of operation, mean (SD)	4.62 (0.6)
Overall satisfaction, mean (SD)	4.61 (0.6)
Security, mean (SD)	4.55 (0.6)
Performance (accurate results, quick response), mean (SD)	4.55 (0.6)
Provide popular science information, mean (SD)	4.55 (0.7)
Blood glucose meter	
Ease of operation, mean (SD)	4.64 (0.6)
The stability with APP connection, mean (SD)	4.62 (0.6)
Overall satisfaction, mean (SD)	4.58 (0.7)

### Adverse events

No serious adverse events were reported from the time of enrollment to the completion of this study. Incidents of moderate ketoacidosis and hypoglycemia were infrequent and showed no significant differences among the groups over the 6-month period (**Table 6**). Additionally, there were no reported or detected deaths, direct study-related adverse events, or severe hypoglycemic episodes.

**Table 6:** Moderate ketoacid and hypoglycemic events.

	G1 ^a^(*n*=50)	G2^b^(*n*=58)	G3^c^(*n*=53)	G4^d^ (*n*=59)	*P* value
Acute complications^e^					1.000
Moderate ketoacid, n (%)	1 (2.0)	0 (0.0)	0 (0.0)	1 (1.7)	
Hypoglycemia, n (%)	14 (28.0)	13 (22.4)	11 (20.8)	16 (27.1)	

^a^G1, usual care; ^b^G2, telephone follow-up; ^c^G3, App; ^d^G4, App & telephone follow-up; ^e^Per participant who experienced acute complications 6 months after intervention.

## Discussion

### Principal findings

This study utilized a factorial design to investigate a mobile app–based diabetes self-care intervention for hospital patients with T2DM over a 6-month period. While there was an overall increase in HbA_1c_ attainment rates across all four groups from baseline, the changes in attainment rates did not significantly differ among the groups over the six months. Upon intervention, we observed that the diabetes app (G3 and G4) appeared to improve HbA1c attainment rates at the 3- and 6-month marks. The app automatically gathers patients’ health data and offers robust interactive feedback features. These include automated analyses and recommendations following blood glucose monitoring, as well as generating weekly and monthly blood glucose reports. These features encourage patients to actively engage in managing their condition, thereby facilitating personalized diabetes management. Studies have shown that a high baseline HbA_1c_ level is an independent predictor of a decline in blood glucose levels (13). Specifically, the higher the baseline HbA_1c_ level, the greater the potential for reduction. In this study, the baseline HbA_1c_ levels of participants ranged from 8.0 to 8.4%, which is lower than those reported in the studies by Gunawardena KC (14) and Xu HW (15). This difference may explain why the HbA_1c_ compliance rates in G3 and G4 were slightly higher than that of other groups in our study, although the differences were not statistically significant.

Following the intervention, there was a statistically significant effect on SBP. Factorial analysis indicated that the app effectively reduced patients’ SBP, consistent with findings by Gong K (16). However, another study reported no significant impact of a smartphone app on BP (17). The Dnurse app is designed within a Chinese cultural framework and, while not specifically targeting patients with hypertension, it creates personalized diet and exercise plans. Additionally, it facilitates interactions in a community setting, encouraging the adoption of a healthy lifestyle. Numerous studies have validated the efficacy of lifestyle interventions in lowering BP and preventing or delaying hypertension (18, 19). Research has also demonstrated a positive correlation between HbA_1c_ and BP (20). In this study, HbA_1c_ levels decreased across all groups after intervention, which could contribute to the reduction in BP. Although the post-intervention BP in all groups met the target for BP control in T2DM in China, HbA_1c_ levels did not reach the set target (1). This suggests that reductions in BP are more pronounced than those in HbA_1c_, possibly due to the greater sensitivity of BP changes. To enhance the study’s reliability and further examine the effect on BP, ambulatory blood pressure monitoring may be considered.

SMBG is a vital component of diabetes self-management. It enables patients to better comprehend their disease status and serves as a crucial foundation for timely adjustments in diet, exercise, and medication regimens during medical consultations. Previous research has highlighted the effectiveness of two intervention methods—phone follow-up (21) and mobile apps (22)—in enhancing patients’ glucose self-monitoring behavior. In this study, although the SMBG compliance rate in the two phone follow-up groups was higher than that in those without such follow-ups, no significant differences in compliance rates were observed among the four groups. This suggests that both weekly phone follow-ups and app reminder features have similar impacts on SMBG compliance. However, regular phone follow-ups can increase labor costs and potentially affect the sustainability of the intervention, while app-based intelligent reminder features offer a more cost-effective solution. To enhance the effectiveness of the app’s intelligent reminders, future improvements may be made in the blood glucose monitoring features, such as introducing system rewards for consistent monitoring or integrating phone interventions into automated customer service.

### Comparison with prior work

Prior studies have demonstrated that diabetes management apps can enhance blood glucose control in patients with diabetes (23–25). However, the majority of these studies have been conducted in developed countries or regions. Research by Lim SL (6) and Yang Y (26) indicated that using mobile apps for diabetes management significantly improved blood glucose control and also reduced BP and weight. Nonetheless, both studies were carried out in economically advanced countries, leaving the efficacy of these apps in less developed regions uncertain. Unlike other studies (27, 28), our research did not exclude older participants, resulting in a more representative sample of the T2DM population. For instance, Kang S’s study (29), which excluded patients over 45, found that app-based self-management support helps young and middle-aged diabetic patients achieve target HbA_1c_ levels. Moreover, some studies suggest that app usage is more effective in young patients compared to the elderly (23, 26). In our study, nearly one-third of participants were over 60. Given that older patients typically have lower awareness and acceptance of new technologies than younger ones, an app that is effective for middle-aged participants may not necessarily yield the same results in an older population. This aligns with the current demographic trends in diabetes, as the aging population is contributing to a rise in the number of elderly diabetic patients (30, 31). This indicates a potential to develop apps tailored to different user groups, such as simplifying the user interface and increasing font sizes to make them more suitable for older adults.

Regarding the research design, we utilized a factorial design to achieve two main objectives. Firstly, this approach allows us to verify the effectiveness of using an app for blood glucose management. Secondly, by comparing the impact of the app’s intelligent reminders with manual phone follow-ups on patients’ blood glucose monitoring compliance rates, we aim to identify the most effective method to enhance patient compliance in blood glucose monitoring.

### Limitations

Although this study employs a rigorous factorial design with participants recruited from two large medical centers in China, there are some design limitations that should be taken into account when interpreting the results. We used convenience sampling in this study, which may limit the representativeness of the sample. Consequently, the generalizability of the findings to other T2DM populations in China needs further investigation. While we cannot entirely prevent participants in G1 and G2 from using other diabetes-related apps during the study, they will not have access to the Dnurse App, which is central to this trial. Additionally, the intervention lasted only six months, so the long-term effects of the Dnurse App on participants’ self-management need further exploration.

## Conclusions

In this study, the HbA_1c_ attainment rates in G3 and G4 were generally higher than those in the other groups after both three and six months. Notably, the BP control improvement in G3 and G4 after three months showed a significant difference from that in G1 and G2. We found no statistical difference between the blood glucose monitoring functionalities of the app and phone follow-up. However, participants expressed a very high level of satisfaction with the Dnurse App. Our results indicate that the enhanced metabolic control observed in the app-user groups can be attributed to the app’s Intelligent Decision Support System, which interacts with users in real time and promotes patient autonomy. Based on our findings, we conclude that telemedicine is an effective and safe approach for T2DM patients.

## Author’s note

This work was presented at the International Diabetes Federation Western Pacific Region Congress 2023 15th Scientific Meeting of the Asian Association for the Study of Diabetes, July 21-23, 2023. as an oral presentation (No. WOEC-02-5).

## Data Availability

The original contributions presented in the study are included in the article/supplementary material. Further inquiries can be directed to the corresponding authors.
